# Laparoscopic Proximally Extended Colorectal Resection With Two-Stage Turnbull-Cutait Pull-Through Coloanal Anastomosis for Late Complications of Chronic Radiation Proctopathy

**DOI:** 10.3389/fsurg.2022.845148

**Published:** 2022-04-25

**Authors:** Yanjiong He, Zuolin Zhou, Xiaoyan Huang, Qi Guan, Qiyuan Qin, Miaomiao Zhu, Huaiming Wang, Qinghua Zhong, Daici Chen, Hui Wang, Lekun Fang, Tenghui Ma

**Affiliations:** ^1^Department of Colorectal Surgery, Sun Yat-sen University Sixth Affiliated Hospital, Guangzhou, China; ^2^Guangdong Provincial Key Laboratory of Colorectal and Pelvic Floor Diseases, Sun Yat-sen University Sixth Affiliated Hospital, Guangzhou, China; ^3^Department of Pharmacy, Sun Yat-sen University Sixth Affiliated Hospital, Guangzhou, China; ^4^Guangdong Institute of Gastroenterology, Sun Yat-sen University Sixth Affiliated Hospital, Guangzhou, China; ^5^Department of Clinical Laboratory, Sun Yat-sen University Sixth Affiliated Hospital, Guangzhou, China

**Keywords:** chronic radiation proctopathy, laparoscopic proximally extended colorectal resection, Turnbull-Cutait pull-through, two-stage coloanal anastomosis, PE-Bacon

## Abstract

**Background:**

Chronic radiation proctopathy (CRP) is a common complication after radiation therapy for pelvic malignancies. Compared with diversion surgery, resection surgery removes the damaged tissue completely to avoid the risks of recurrence and improve patients' outcome. Hence, resection surgery could be an optimal surgical approach when CRP is complicated by late complications. This study aimed to describe a modified surgical procedure of resection surgery and report its preliminary efficacy and safety in treating patients with CRP with late complications.

**Methods:**

We retrospectively reviewed the patients who were diagnosed with CRP with late complications and underwent the modified surgical procedure of laparoscopic proximally extended colorectal resection with two-Stage Turnbull-Cutait pull-through coloanal anastomosis (PE-Bacon) between November 2019 and October 2020 in the Sixth Affiliated Hospital of Sun Yat-sen University.

**Results:**

A total of 15 patients were performed the modified laparoscopic procedure of PE-Bacon, of which 1 patient underwent conversion from laparoscopic to open operation for intraoperative massive hemorrhage. Overall, the major (Clavien-Dindo III-V) postoperative complications occurred in 1 patient, anastomotic leakage was observed in 2 (13.3%) patients, and anastomotic stricture was observed in 4 (26.7%) patients. No patient had to be reoperated and died. Up to now, at the average follow-up of (524.40 ± 108.39) days, the preoperative symptoms of 93.3% (14/15) patients were relieved, with nine patients achieved complete remission, five patients only suffered minor symptoms. Because of the progression of radiation uropathy, one patient still had a vesicovaginal fistula as pre-operative complication. Colostomy reversal has been performed on 8 (53.3%) patients at an average postoperative duration of 299.5 ± 92.68 days, among whom only 2 patients suffered from major Low Anterior Resection Syndrome (LARS) until now.

**Conclusions:**

Laparoscopic PE-Bacon surgery is a safe and feasible surgical procedure for late complications of CRP with low morbidity and high symptom remission rate.

## Introduction

Chronic radiation proctopathy (CRP) is a common complication after radiation therapy for pelvic carcinomas, and its primary clinical manifestations are diarrhea, rectal bleeding, abdominal pain, fecal incontinence, and anal pain (1, 2). The chronic phase of radiation proctopathy is characterized by inflammation, irreversible fibrosis, and occluding vasculitis, and platelet-derived growth factor C signaling was found to be activated in our previous study (3). Due to radiotherapy, the damaged bowel has a poor wound healing (4), which is progressive and can lead to fistula, perforation, stricture, severe bleeding and deep ulceration, severely affecting patients' quality of life (5, 6). When it leads to above-mentioned late complications, surgical intervention is always required, and resection of diseased segment of the bowel and diversion are the main type of surgery (7, 8).

Up to now, there is sparse agreement on the types of surgery. Resection of the damaged bowel removes the damaged tissue to avoid the risks of recurrence and annoying symptoms and improve patient outcome, which could be an ideal surgical approach for CRP (9, 10). However, because of high incidence of postoperative complications and mortality, resection of the diseased bowel may not be the optimal surgical options in the treatment of CRP (11–14). However, with the advances of surgical techniques and perioperative care, the morbidity and mortality of resection surgery has been decreased significantly (15). More attention should be paid on the resection of the affected bowel and reconstruction of intestinal continuity for its potential to improve the quality of life of patients and long-term survival (16–20).

Anastomosis with at least one end of bowel without radiation damage can greatly reduce postoperative anastomotic leakage rate and mortality (21). And in Bacon surgery, primary anastomosis is not performed, and the anastomotic tension markedly reduced and the blood supply of anastomosis can be judged intuitively to improve the quality of anastomosis in the second stage of intestinal anastomosis to decrease the anastomotic leakage rate (22). Combining the advantages of proximally extended resection and two-stage anastomosis to minimize potential complications and maximize the therapeutic efficacy, in this study, the included patients with late complications of CRP were performed modified procedures of laparoscopic proximally extended colorectal resection with two-stage Turnbull-Cutait pull-through coloanal anastomosis (PE-Bacon in short). And PE-Bacon surgery was described, and its preliminary efficacy and safety in treatment of late complications of CRP were evaluated.

## Materials and Methods

### Patients

The study was approved by the ethical committee of the Sixth Affiliated Hospital of Sun Yat-sen University (No. 2021ZSLYEC-194) and was conducted in according with the provisions of the World Medical Association's Declaration of Helsinki of 1995 (revised in Tokyo, 2004). Informed consent was waived due to this is a retrospective study. Patients eligible for inclusion were patients were confirmed CRP with late complications such as fistulas, perforations, strictures, severe bleeding, and ulceration; Patents underwent the surgical procedure of PE-Bacon. The exclusion criteria included patients with incomplete clinical data. In the present study, a total of 15 patients were included in the Sixth Affiliated Hospital of Sun Yat-sen University between November 2019 and October 2020.

### Operative Procedure

#### Preoperative Preparation

In the absence of intestinal obstruction and severe rectal symptoms, full mechanical bowel preparation and oral antibiotics were necessary. Full mechanical bowel preparation was accomplished with oral polyethylene glycol solution in afternoon or evening before surgery. Oral antibiotics follows mechanical bowel preparation in the afternoon or evening before surgery, and three repeated doses of oral gentamicin (80,000 unit) and metronidazole (0.4 gram) were given orally over a period of ~10 h.

#### Surgical Techniques

The surgical procedure consisted of two stages: (a) laparoscopic proximally extended colorectal resection of the diseased bowel with Turnbull-Cutait pull-through coloanal anastomosis and (b) the second-stage coloanal anastomosis.

##### First Stage

The patients were placed in the modified Lloyd-Davies position. Bilateral ureteric stents were routinely placed for exposing bilateral ureters intraoperatively. The standard five-port method was used and trocars were positioned at right and left upper quadrants, right and left lower quadrants, and supraumbilical area.

**Figure 1:**
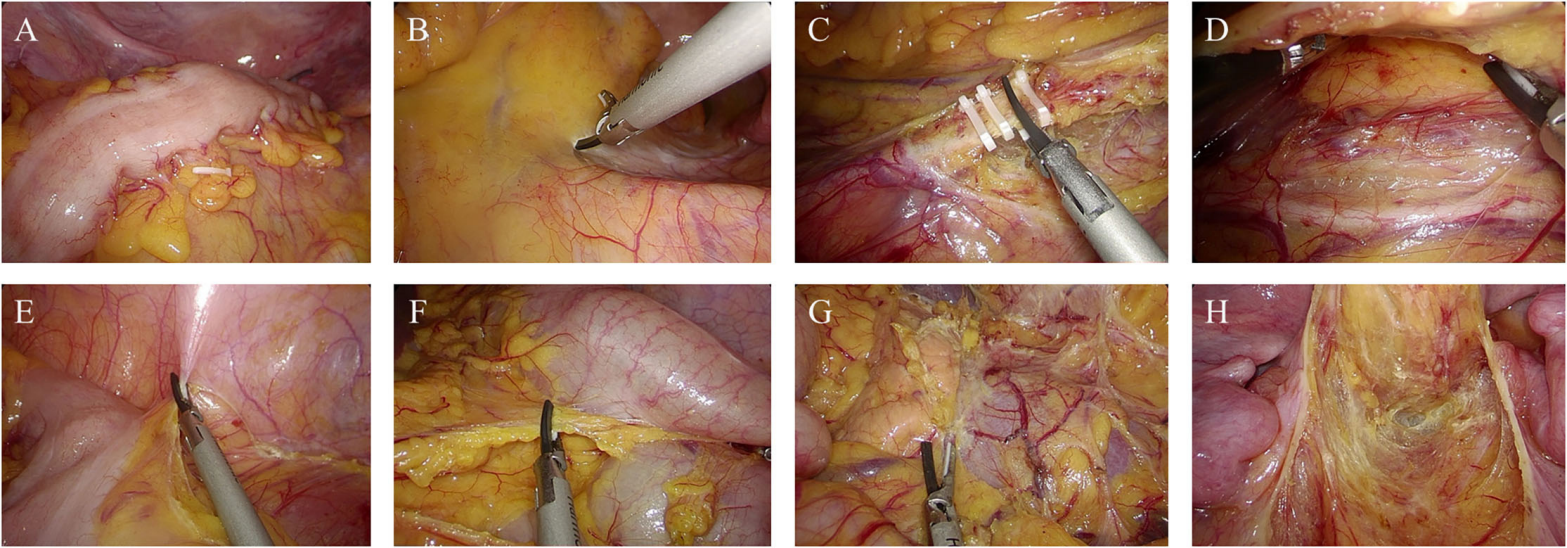
Abdominal phase of the first stage of PE-Bacon procedure. **(A)** Mark at the proximal site of non-irradiated colon. **(B)** Incise the mesentery of sigmoid along the sacral promontory. **(C)** Ligate inferior mesenteric vessels. **(D)** Expand the Toldt's fascia. **(E,F)** Mobilize the colon toward the splenic flexure. **(G)** Dissect the phrenicocolic ligament and splenocolic ligament. **(H)** Mobilize the rectum.

**Initial Exploration:** Careful evaluation was performed to make sure that there were no primary tumor recurrence or metastasis and to identify the range and degree of radiation intestinal injury. After the evaluation, a hemo-lock clip was marked at the distal site of non-irradiated colon (Figure 1A). If the abdominal adhesion was severe, conversion of laparoscopic procedure to open surgery might be necessary to reduce the surgical difficulty.

#### Abdominal Phase

**Mobilization of sigmoid:** The sigmoid was incised along the sacral promontory and retrosigmoid space was expanded to left paracolic gutter (Figure 1B). Ligation and division of inferior mesenteric artery and inferior mesenteric vein were performed (Figure 1C).

**Mobilization of the left colon:** The peritoneum of left paracolic gutter was incised and then dissected along the white line of Toldt fascia toward the splenic flexure (Figure 1D). Expand the space and dissect phrenicocolic ligament and splenocolic ligament until the splenic flexure is completely mobilized (Figures 1E–G).

**Mobilization of the rectum:** The retrorectal space is developed along the retrosacral fascia with sharp dissection to pelvic floor. The anterior dissection of rectum is performed along the surface of Denonvilliers fascia (Figure 1H).

#### Perineal Phase

**Figure 2:**
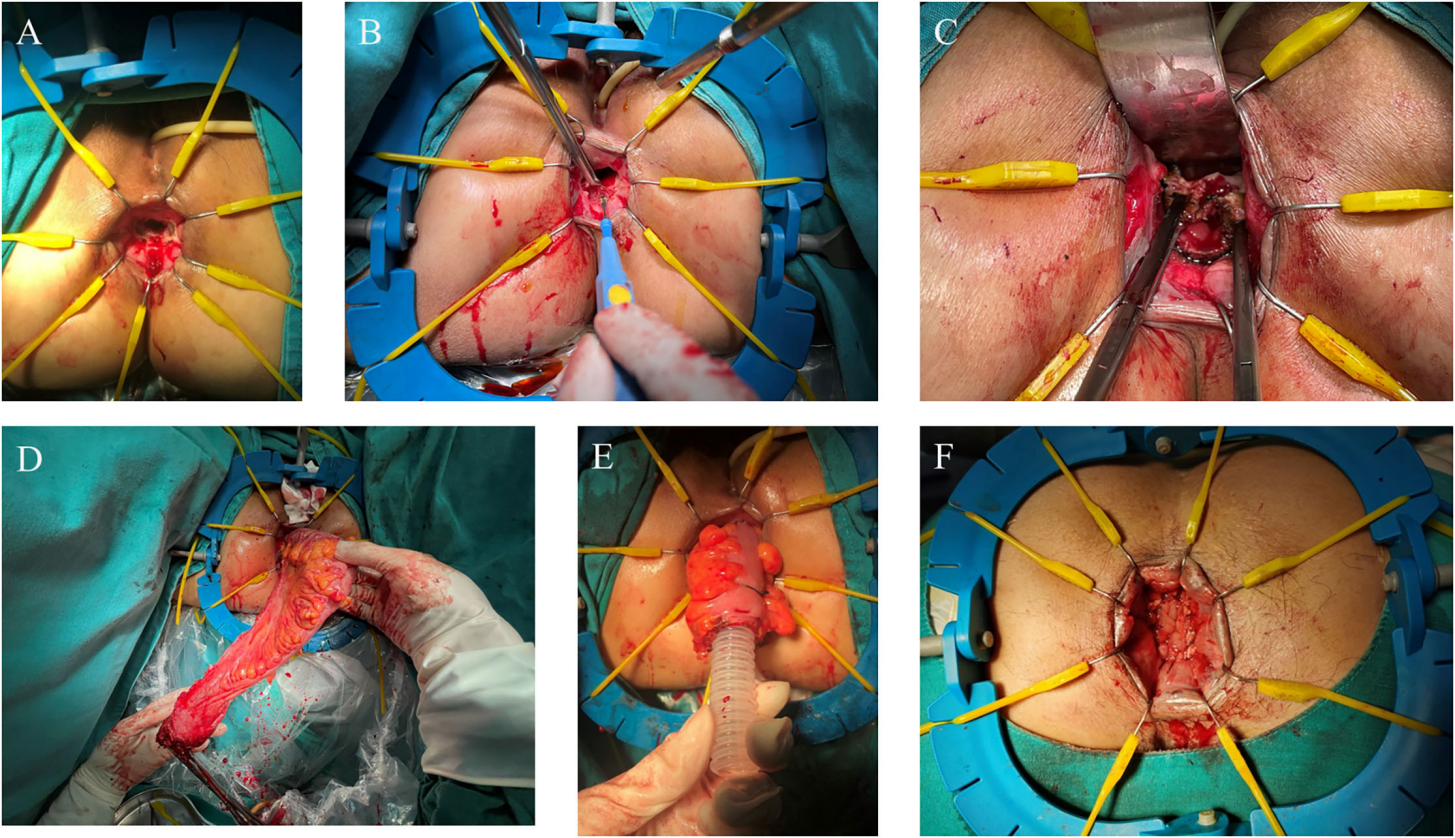
Perineal phase of PE-Bacon procedure. **(A)** Expose the anal canal; **(B)** dissect the rectum at the level of 1 cm from the distal margin of the rectal lesion; **(C)** dissect the rectum along the intersphinteric plane; **(D)** pull through the colon and rectum and transect the diseased bowel; **(E)** first-stage coloanal anastomosis. **(F)** second-stage coloanal anastomosis.

A Lone Star Retractor was used to facilitate exposure of the anal canal (Figure 2A). The rectum was transected at the level of 1 cm from the distal margin of the rectal lesion, and then the pelvic dissection was performed in the intersphinteric plane (Figures 2B,C). The tension and blood supply of the proximal colon were evaluated before the distal colon and rectum were pulled through the anal canal (Figure 2D). After the pull-through of proximal colon, the diseased bowel was removed at the level of hemo-lock clip, leaving a 5–7 cm of colonic stump. 3-0 Vicryl sutures were placed along the circumference of anal canal to avoid the retraction of the colonic stump (Figure 2E).

**Drains and protective transverse colostomy:** After the resection and the pull-through, a drain was placed in pelvic. Protective transverse colostomy was performed.

##### Second Stage

The second-stage coloanal anastomosis was scheduled 1–2 weeks after the first stage. Patients were placed in the fold position under epidural anesthesia. The exteriorized colonic stump was resected and the coloanal anastomosis was performed between anal canal and colon (Figure 2F).

##### Technical Tips

If the mesorectum is so thick or the pelvic space is so limited that the countertraction is insufficient and the surgical difficulty is high, we can transect the rectum ahead of schedule to gain more surgical space. When the adhesion in abdominal cavity is too severe to expand the right space, we can sacrifice part of rectal wall or mesorectum to avoid the surgical injury by mistake.

##### Outcome Measures

The following clinical data was collected retrospectively. Patient characteristics such as age, primary carcinomas, type of complication, duration of symptoms, previous treatment, and ASA grade. Surgery details included surgery type, operative time, intraoperative blood loss, and total length of resected bowel. Postoperative data included postoperative complications and postoperative recovery. Postoperative complications were classified by Clavien-Dindo classification system, and the major complications were defined as grade III–V complications (23).

### Postoperative Follow-Up

Follow-up was scheduled after 1, 3, and 6 months postoperatively such as digital rectal examination and quality-of-life assessment. Colonoscopy, pelvic MRI, rectal defecography, and anorectal manometry measurement were performed to assess whether colostomy reversal can be performed in 6 months following the surgery. To assess bowel function, Low Anterior Resection Syndrome (LARS) questionnaire was used to evaluate the severity of LARS in 6 month following colostomy reversal (24). According to the LARS score, the severity of LARS was divided into “No LARS” (0 to 20 points), “minor LARS” (21 to 29 points), and “major LARS” (30 to 42 points). The quality of life was assessed using EORTC QLQ-C30 questionnaire (25), which covered five functional scales, three symptom scales, six individual symptoms, and a global health index.

### Statistical Analysis

Results were presented as proportions (percentage) for categorical variables, and for continuous variables, results were present as mean ± SE or median (range). The comparison of the quality of life between patient underwent colostomy reversal and patients failed to reverse stoma was performed using the *t*-test. Statistical significance was set at *p* < 0.05.

## Results

### Patient Characteristics

The demographics of the patients are presented in Table 1. A total of 15 patients (all women) underwent the PE-Bacon procedure were included in this study. Among them, 8 (53.3%) patients were complicated with rectovaginal fistula, 3 (20.0%) patients were complicated with rectal stricture, and 2 (13.3%) patients were complicated with deep rectal ulcer and rectal stricture.

**Table 1:** Demographics of patients underwent the surgical procedure.

**Demographics**	**No. of patients (%)**
Average age (mean±SD, years)	52.0 ± 6.9
**BMI (kg/m**^**2**^**) prior to surgery**, ***n*** **(%)**	
<18.5	4 (26.7)
≥18.5	11 (73.3)
**Primary carcinoma**, ***n*** **(%)**	
Cervical carcinoma	14 (93.3)
Endometrial carcinoma	1 (6.7)
Abdominal surgery history, *n* (%)	3 (20.0)
**Comorbidity**	
Hypertension	3
**Late complications**, ***n*** **(%)**	
Rectovaginal fistula	8 (53.3)
Rectal stricture	3 (20.0)
Deep rectal ulcer and rectal stricture	2 (13.3)
Deep rectal ulcer	1 (6.7)
Deep rectal ulcer and sigmoid perforation	1 (6.7)
Duration of symptoms (months)	6 (1–58)
The median latency time of symptoms (months)	5 (1–80)
**Symptoms related to CRP**	
Hematochezia	14
Anal pain	11
Rectal fistula	8
Anal bloating	4
Diarrhea	3
Abdominal pain	2
**Previous treatment to CRP**, ***n*** **(%)**	
Enema	8 (53.3)
Argon plasma coagulation	3 (20.0)
Diversion	7 (46.7)
**Preoperative ASA grade**, ***n*** **(%)**	
II	14 (93.3)
III	1 (6.7)

*ASA, american association of anesthesiologists; BMI, body mass index*.

### Perioperative Characteristics

Table 2 shows the perioperative characteristics and postoperative complications of the included patients. Among these patients, 14 patients underwent laparoscopic surgery, and 1 patient underwent conversion from laparoscopic to open operation1 patient underwent conversion from laparoscopic to open operation because of intraoperative massive hemorrhage caused by the splenocolic ligament tear. The average operative time was (267.40 ± 43.95) min, and the median intraoperative blood loss was 80 (50–2,000) ml. Because of the splenocolic ligament tear, 1 patient had intraoperative massive hemorrhage and needed blood transfusion. The average total length of resected diseased bowel was (21.70 ± 8.26) cm in the two-stage schedule.

**Table 2:** Perioperative characteristics and postoperative complications.

**Variables**	**No. of patients (%)**
**Surgery type**, ***n*** **(%)**	
Laparoscopic surgery	14 (93.3)
Conversion to open surgery	1 (6.7)
Operative time (min), mean (±SD)	267.40 (±43.95)
Intraoperative blood loss (ml), median (range)	80 (50–2000)
total length of resected bowel (cm), mean (±SD)	21.70 (±8.26)
Time to first passage of flatus (days), median (range)	2 (1–3)
Postoperative defecation time (days), median (range)	2 (1–7)
drainage tube removal time (days), median (range)	4 (2-5)
Postoperative hospital stay (days), mean (±SD)	12.73 (±4.08)
Major postoperative complications, *n* (%)	1 (6.7)

The median postoperative hospital stay was (12.73 ± 4.08) days. The major (grade III-V) postoperative complications at day 30 occurred in 1 patient. No patient had to be reoperated and died because of postoperative complications at day 30. The median time to first flatus was 2 (1–3) days, and the median defecation time was 2 (1–7) days. The median drainage tube removal time was 4 (2–5) days.

### Follow-Up

The results of follow-up are showed in Table 3. The average follow-up was (524.40 ± 108.39) days. Vesicovaginal fistula was observed in 1 patient for the progression of radiation uropathy.

**Table 3:** The follow-up of patients underwent the surgical procedure.

**Variables**	**No. of patients (%)**
Follow-up period (days), mean (±SD)	524.40 (±108.39)
**Complication related to anastomosis**, ***n*** **(%)**	
Anastomotic leakage	2 (13.33)
Anastomotic stricture	4 (26.67)
**Symptoms related to CRP**, ***n*** **(%)**	
Complete remission	9 (60)
Minor symptoms related to CRP	5 (33.3)
Minor anal pain	3
Anal bloating	1
Rectovaginal fistula	1
Abdominal pain	2
Symptoms related to anastomotic leakage	1 (6.7)
Colostomy reversal, *n* (%)	8 (53.3)
Time to colostomy reversal (days), mean (±SD)	299.50 (±92.68)
**Low anterior resection syndrome**, ***n*** **(%)**	
No LARS	1 (12.5)
Minor LARS	5 (62.5)
Major LARS	2 (25.0)
Maximum resting pressure (mmHg), median (range)	33.40 (26.30–60.00)
Maximum squeeze pressure (mmHg), median (range)	92.60 (47.90–119.00)

Anastomotic leakages were observed in two patients. Anastomotic stricture was observed in four patients. Among them, anastomotic stricture plasty was required in two patients for anastomotic stricture and dilatation of the anus was performed in other two patients.

Complete remission of symptoms was achieved by 9 (60%) patients. Only 5 (33.3%) patients suffered minor symptoms related to CRP including minor anal pain and abdominal pain. Symptoms related to anastomotic leakage and rectovaginal fistula are suffered by 1 (6.7%) patients.

Up to now, colostomy reversal has been performed on 8 (53.3%) patients at average postoperative 299.5 ± 92.68 days. After 296.7 ± 107.2 days of average interval between colostomy reversal and surgery, five patients had minor LARS, and two patients had major LARS. At the same time, anorectal manometry showed that the median maximum resting pressure was 33.40 (26.30–60.00) mmHg, and the median maximum squeeze pressure was 92.60 (47.90–119.00) mmHg.

To display the treatment details of all patients fully, details of surgery, perioperative recovery, and follow-up are given in Table 4.

**Table 4:** Details of surgery, perioperative recovery, and follow-up.

**No**.	**Age**	**Preoperative symptoms**	**Surgical indication**	**Surgical types**	**Duration(min)**	**Blood loss (ml)**	**Blood transfusion**	**Postoperative hospital stay (d)**	**Perioperative major complications**	**Anastomotic leak**	**Anastomotic stricture**	**Colostomy reversal**	**Interval between colostomy reversal and surgery (d)**	**Fail to reverse stoma**	**Postoperative symptoms**	**LARS**	**Interval between latest follow-up and surgery (or as colostomy reversal conducted) (d)**
1	Y54	Hematochezia, anal pain, rectal fistula, diarrhea	Rectovaginal fistula	Laparoscopic	256	100	No	14	None	No	No	Yes	380	——	Complete remission	Minor	719 (339)
2	Y42	Hematochezia, diarrhea, abdominal pain	Rectal stricture	Laparoscopic	255	50	No	9	None	No	Yes	Yes	201	——	Complete remission	Minor	702 (501)
3	Y42	Hematochezia, rectal fistula, anal bloating	Rectovaginal fistula	Laparoscopic	274	100	No	12	None	No	Yes	Yes	445	——	Complete remission	Major	681 (236)
4	Y53	Hematochezia, anal pain, anal bloating, abdominal pain	Rectovaginal fistula	Laparoscopic	233	100	No	13	None	No	No	No	——	Tumor recurrence	Anal bloating, inor abdominal pain	——	620
5	Y53	Hematochezia, anal pain, rectal fistula, abdominal pain	Rectovaginal fistula	Laparoscopic	305	100	No	13	None	Yes	No	No	——	Anastomotic leak	Rectovaginal fistula	——	620
6	Y62	Hematochezia, anal pain	Deep rectal ulcer and sigmoid perforation	Conversion to open surgery	315	2000	Yes	23	Hemorrhagic shock	No	No	No	——	Patient's refusal	Complete remission	——	613
7	Y50	Hematochezia, anal pain	Deep rectal ulcer and rectal stricture	Laparoscopic	226	80	No	14	None	No	No	Yes	223	——	Minor anal pain	No	604 (381)
8	Y64	Hematochezia, anal pain, diarrhea, abdominal pain	Rectovaginal fistula	Laparoscopic	235	50	No	11	None	No	No	Yes	349	——	Abdominal pain	Minor	558 (209)
9	Y51	Hematochezia, rectal fistula	Rectovaginal fistula	Laparoscopic	325	50	No	10	None	No	No	Yes	351	——	Complete remission	Major	548 (197)
10	Y56	Hematochezia, anal bloating	Rectal stricture	Laparoscopic	208	100	No	10	None	No	No	Yes	235	——	Complete remission	Minor	540 (305)
11	Y62	Hematochezia, anal pain, anal bloating	Rectovaginal fistula	Laparoscopic	271	60	No	19	None	No	No	No	——	Patient's refusal	Complete remission	——	484
12	Y57	Hematochezia, anal pain, abdominal pain	Rectal stricture	Laparoscopic	214	50	No	9	None	Yes	Yes	No	——	Anastomotic leak and stricture	Minor anal pain	——	445
13	Y55	Anal pain	Rectovaginal fistula	Laparoscopic	235	50	No	16	None	No	Yes	Yes	212	——	Minor anal pain	Minor	417 (205)
14	Y50	Hematochezia, anal pain	Deep rectal ulcer and rectal stricture	Laparoscopic	347	100	No	9	None	No	No	No	——	Poor anal function	Complete remission	——	409
15	Y44	Hematochezia, anal pain	Deep rectal ulcer	Laparoscopic	312	50	No	9	None	No	No	No	——	Patient's refusal	Complete remission	——	382

Quality of life was assessed using EORTC QLQ-C30 questionnaire. Patients had good scores in function subscales and symptom subscales in our series. The results of assessment are presented in Table 5.

**Table 5:** Evaluation of quality of life in patients underwent the PE-Bacon.

**Domain**	**Colostomy reversal (M±SD)**	**Fail to reverse stoma (M±SD)**	**Total (M±SD)**	***p***
**Function subscales**				
Physical function	90.83 ± 15.91	87.62 ± 13.57	89.33 ± 14.43	0.68
Role function	81.25 ± 27.37	83.33 ± 16.67	82.22 ± 22.24	0.86
Emotional function	88.54 ± 13.32	90.4 ± 15.53	89.44 ± 13.89	0.80
Cognitive function	100	100	100	
Social function	85.42 ± 18.77	85.71 ± 15.00	85.56 ± 16.50	0.97
Global health/Quality of life	63.54 ± 13.32	53.57 ± 6.56	58.88 ± 11.56	0.096
**Symptom subscales/items**				
Fatigue	22.22 ± 14.55	19.05 ± 21.00	20.74 ± 17.25	0.74
Nausea/vomiting	0	0	0	
Pain	12.50 ± 23.15	21.43 ± 23.00	16.67 ± 22.71	0.47
Dyspnea	8.33 ± 23.57	9.52 ± 16.27	8.88 ± 19.78	0.91
Insomnia	37.50 ± 37.53	42.86 ± 31.71	40.00 ± 33.81	0.77
Appetite loss	0	14.29 ± 26.23	6.67 ± 18.69	0.15
Constipation	0	0	0	
Diarrhea	0	0	0	
Financial difficulties	25.00 ± 15.43	42.86 ± 16.27	33.33 ± 17.82	0.048

## Discussion

Radiotherapy is an essential therapeutic method in the treatment of pelvic malignancies, and about 35–61% of patients with pelvic malignancies receive radiotherapy (26). Even though radiotherapy prolongs the lifetime of patients, it also has great damage to pelvic organs such as bowels, which has negative effect on patients' global health (27). CRP refers to radiation-induced injury to rectum occurring at least 3 months after completion of radiotherapy (28). About 5–20% of patients receiving radiotherapy may suffer CRP, and only 55% of them visited to hospital for persistent symptoms related to CRP (18). Moreover, surgical intervention is indicated in one-third of patients with CRP who were complicated with late complications associated with CRP such as fistulas, perforations, strictures, severe bleeding, and ulceration (18).

The surgery types of late complications of CRP involve resection of the injured bowels and diversion. But up to now, the optimal choice of the surgical treatment remains controversial. A meta-analysis by McCrone et al. concluded that diversion alone did not remove the damaged tissue and leaves the patient at risk of recurrent complications, but the surgical risk was higher in resection of the diseased bowel (8). And another meta-analysis by Zhou et al. also concluded that compared with diversion alone, resection surgery had higher risk of complications and comparable mortality, but had higher symptom remission rate and had higher rate of restoring intestinal continuity (29). The early studies reported that resection had a high morbidity and mortality, but most of them were retrospective and were limited by their small sample sizes (11, 12). Kimose et al. performed a retrospective analysis on the largest series of 131 patients requiring surgery for CRP, and the results conducted that the overall outcome for resection group was better than that for diversion group (30). Similar results can be seen in other studies (15). In our series, 7 (46.7%) patients who have underwent diversion previously still needed to undergo resection because of the recurrence and progression of CRP. Total 13 (86.7%) patients achieved complete remission of symptoms or only suffered minor symptoms. Meanwhile, only one patient experienced the major postoperative complications and no patient died of postoperative complications. With the advance of surgical technique and the improvement of surgical procedures, resection of the diseased bowel seems to be feasible and safe in treating late complications of CRP, and meanwhile have a high remission rate.

From our perspective, our surgical procedures present several superiorities in treating late complications of CRP.

Firstly, the aim of proximally extended resection is to make sure that the proximal end of anastomosis is non-irradiated. Previous studies have revealed that avoiding the anastomosis of radiated bowel could reduce incidence of anastomotic leak and mortality and achieved good functional results (31–34). Galland et al. reported that for use of non-irradiated bowel for at least one end of an anastomosis, the rate of anastomotic leak decreased from 51.8% (14/27) to 7.1% (1/14) and the mortality decreased from 37% (10/27) to 0 (35). The clinical target volumes for radiation therapy in pelvic malignancies cover pelvic cavity, so the anal canal and the rectum are irradatiated, and the sigmoid is potentially irradiated. To minimize the radiation injury of the anastomotic site, we performed the proximally extended resection above the level of the upper boundary of pelvis and the close dissection of the diseased bowel downward until the pelvic floor was reached.

The second superiority of the surgical procedure is represented by pull-through and the two-stage coloanal anastomosis. Between the two surgical stages, the adhesions between colon and anal canal have been formed, and blood supply of the distal bowel also can be observed. After waiting 1–2 weeks, amputation of the exteriorized segment with the colonanal anastomosis could be performed safely to avoid the anastomotic leakage and pelvic abscess formation. Ramzi et al. reported that compared to immediate coloanal anastomosis, the incidence of abscess formation and anastomotic leakage was lower after abdominoperineal pull-through with coloanal anastomosis for various complex anorectal condition including persistent leakage and rectovaginal fistula (3 vs. 7%) (36). Similar result can be seen in other studies (37, 38). The advantage of the pull-through and the two-stage coloanal anastomosis also seems to be confirmed in our study. In our study, only 1 patient suffered anastomotic leakage.

Last but not least, laparoscopic surgery has advantages of minimal invasive surgery. According to the study by Wang et al. laparoscopic surgery for radiation enteritis can decrease operative time, intraoperative blood loss, and postoperative recovery time with a relative low conversion rate (39). In our series, the conversion rate is relatively low, because the intestinal adhesion mainly is located at the pelvic cavity, and there is adequate space for establishing pneumoperitoneum. The application of laparoscope can help us to recognize the anatomic structures to avoid surgical injuries.

Even though the procedures of proximal extended resection and pull-through greatly enhance the safety and feasibility, protective transverse colostomy is necessary in such patients. Previous studies have identified several risk factors for anastomotic leakage such as the nutritional status of patients, anastomotic height, and preoperative radiotherapy (40–42). Similarly, because of poor nutritional status, the intestinal radiation injury, and low anastomosis, the risk of anastomotic leakage is relatively higher in such patients. Protective colostomy can reduce and even avoid severe pelvic infection and abscess, septic shocks related to anastomotic leakage, and avoid reoperation caused by anastomotic leakage. In our series, one patient had anastomotic leakage but didn't undergo reoperation for protective colostomy.

The functional outcomes of our surgical procedure still remain critical in our study because only eight patients underwent colostomy reversal and the follow-up period was not long enough. Among the patients underwent colostomy reversal, only two patients suffered major LARS during more than 1-year follow-up, which was a satisfactory bowel function outcome. And in other similar surgical procedures, satisfactory bowel functional outcomes were stated (36, 43, 44). The quality of life was assessed through EORTC QLQ-C30 questionnaire in our study. Patients had good scores in function subscales and symptom subscales in our series because of the remission of symptoms related to CRP. However, the general health of such patients still needs to be focused on during the long-term follow-up. Interestingly, the quality of life may be not closely associated with the colostomy reversal surgery in our study population.

The present study has several limitations. Firstly, the sample size was not large enough, only 15 patients being enrolled. A larger population study is needed to confirm our results. Secondly, the present study was limited by its retrospective design. Thirdly, the functional outcomes and the quality of life need to be followed up for a longer time. A prospective clinical trail with larger sample size is in progress by our team to confirm our findings in this study.

## Conclusion

The surgical treatment of CRP still remains challenging and controversial due to the characteristic of patients and the etiology. As a theoretically ideal surgical procedure, PE-Bacon seems to be a safe and feasible surgical procedure for late complications of CRP with low morbidity and high symptom remission rate. However, the safety and feasibility need to be proved in a larger sample size and the functional outcomes still need to be followed up.

## Data Availability Statement

The raw data supporting the conclusions of this article will be made available by the authors, without undue reservation.

## Ethics Statement

The studies involving human participants were reviewed and approved by the Ethical Committee of the Sixth Affiliated Hospital of Sun Yat-sen University. Written informed consent for participation was not required for this study in accordance with the national legislation and the institutional requirements.

## Author Contributions

TM, HW, LF, YH, ZZ, and XH contributed to conception and design of the study. TM, ZZ, YH, HW, and QQ conducted the modified surgery of PE-Bacon for the patients with CRP. YH, QG, XH, and ZZ collected the data. YH drafted the article. TM, XH, LF, and HW revised the article. All authors were responsible for analysis and interpretation of the data and contributed to and approved the final manuscript.

## Funding

This study was supported by Sun Yat-sen University Clinical Research 5010 Program (Grant Numbers: 2019021 and 2017008), 2021 Bethune Merck Research Fund for Young and Middle-aged Physicians, the Sixth Affiliated Hospital of Sun Yat-sen University Clinical Research−1010 Program [Grant Number: 1010PY (2020)-48], and Science and Technology Projects in Guangzhou (Grant Number: 202102020009).

## Conflict of Interest

The authors declare that the research was conducted in the absence of any commercial or financial relationships that could be construed as a potential conflict of interest.

## Publisher's Note

All claims expressed in this article are solely those of the authors and do not necessarily represent those of their affiliated organizations, or those of the publisher, the editors and the reviewers. Any product that may be evaluated in this article, or claim that may be made by its manufacturer, is not guaranteed or endorsed by the publisher.
